# Optical Imaging of Cilia in the Head and Neck

**DOI:** 10.3390/jcm14062059

**Published:** 2025-03-18

**Authors:** Elizabeth Lee, Lidek Chou, Zhongping Chen, Brian J. F. Wong

**Affiliations:** 1 Beckman Laser Institute, University of California, Irvine, CA 92697, USA; elizlee0326@gmail.com (E.L.); lidekc@uci.edu (L.C.); z2chen@uci.edu (Z.C.); 2 Department of Otolaryngology-Head and Neck Surgery, University of California, Irvine, CA 92612, USA; 3 Department of Biomedical Engineering, University of California, Irvine, CA 92612, USA

**Keywords:** cilia, ciliopathy, ciliary motion, optical imaging, optical coherence tomography

## Abstract

**Background/Objectives**: Cilia are hair-like organelles with various mechanosensory and chemosensory functions. In particular, motile cilia generate directional fluid flow important for multiple processes. Motile ciliopathies have serious clinical implications, including developmental and respiratory disorders. Evaluating the most suitable imaging methods for studying ciliary structure and function has great clinical significance. **Methods**: Here, we provide an overview of ciliary function, imaging modalities, and applications in ciliopathic diseases. **Results**: Optical imaging has become a crucial tool for studying ciliary structure and function, providing high-resolution, non-invasive imaging capabilities that are valuable for in vivo applications. Optical coherence tomography (OCT) is well suited for the visualization of ciliary anatomy and quantitative studies of microfluidic flow. **Conclusions**: A deeper understanding of ciliary biology can lead to novel approaches in diagnosing, treating, and monitoring ciliopathies, contributing to more effective and individualized care.

## 1. Introduction

Cilia are hair-like microscopic organelles on the surface of most eukaryotic cells that perform a range of mechanosensory and chemosensory functions [1]. Motile cilia generate microfluidic flow in various organ systems. Defects in this fluid flow can have severe disease manifestations [2]. During embryonic development, the embryonic node, a transient organ, contains cilia that help determine right-left asymmetry. Impaired nodal flow can result in situs inversus, a condition in which organs are reversed from their normal positions [3,4]. In the respiratory tract, cilia beat in a synchronized manner to expel mucus out of the lungs. As the mucus traps foreign particles and pathogens that are inhaled during breathing, impairment of the mucociliary clearance mechanism leads to chronic respiratory infections [1]. Ventricles in the brain are lined with cilia that circulate cerebrospinal fluid (CSF), and dysfunctional flow is associated with hydrocephalus [3]. As cilia plays a critical role in numerous sites in the body, dysfunctional cilia and the resulting pathologies are active areas of research. Finding the best imaging technique for the anatomical and functional assessment of cilia to understand how dysfunction leads to various pathologies has clinical significance.

Imaging cilia to study ciliary structure and physiology is complex, with several factors to consider. To effectively study ciliary structure at the nanoscopic scale, imaging techniques must achieve a minimum requisite resolution. In addition to structural analysis, observing the behavior of cilia and their interactions with the surrounding environment requires dynamic monitoring and real-time imaging [3]. Thus, these imaging methods must be non-destructive to cells and tissue to avoid disturbing the functional dynamics of the system [5]. Current studies on ciliary motion rely on ex vivo models, such as biopsy specimens and cell cultures, to study ciliary function. However, ex vivo tissue conditions differ from those in vivo. The specimen preparation process may mechanically and chemically disturb ciliated cells, and the function of cells change over time, potentially compromising the accuracy of measurements [6]. The capabilities of imaging modalities are also a consideration. Ciliary action spans a wide range of length scales. Imaging modalities would need to encompass them all to provide a comprehensive study of ciliary dynamics. Additionally, sufficient speed is needed to capture two critical dynamic processes: the three-dimensional movement of individual cilia and the three-dimensional flow fields generated by cilia [3].

Considering these factors, biomedical optical imaging has shown utility in imaging microfluidic ciliary flow. These are a set of imaging techniques that use light to capture images of biological tissues and structures at various scales. One of the first techniques developed was light microscopy, which uses the absorption and scattering of light by samples to provide contrast. As it has one of the simplest optical setups and relatively high speed, it has been commonly used to image fluid flow. Another modality, fluorescence microscopy, obtains images by detecting emitted light from fluorophores. For ciliary flow imaging, two types are typically used: epifluorescence, with which a large area of a sample is captured, and confocal fluorescence, with which light is collected from a very small focus. Lastly, optical coherence tomography (OCT) is analogous to ultrasound but uses light instead of sound [3]. With features such as high sensitivity, rapid frame rates, real-time imaging, a lack of radiation, low cost, and portability [7], these modalities merit further study.

Relevant metrics to study with these techniques include the airway surface liquid (ASL) depth, the thickness of the periciliary liquid (PCL) depth, the ciliary beat frequency (CBF), and the velocity of mucociliary transport (MCT) [5]. The characterization of ASL, PCL, MCT, and CBF has been conducted ex vivo using various techniques, such as X-Z scanning confocal microscopy (ASL) [8], osmium tetroxide fixation (PCL) [9], particle and radiolabeling for MCT [10], and high frame rate phase–contrast microscopy for quantifying CBF [11]. However, many of these techniques require the addition of exogenous dyes, the destruction of tissue, and separate imaging equipment that prevents the simultaneous acquisition of these parameters during dynamic study [12].

Addressing some of these limitations, OCT has shown promise for quantitative studies of ciliary physiology and cilia-generated fluid flow. OCT has a relatively high resolution (~1–10 μm), intrinsic contrast from scattering, and simultaneous data acquisition at multiple depths without the need to move samples [3]. Additionally, velocimetry techniques can be used with OCT imaging, including Doppler, particle tracking velocimetry (PTV), digital particle image velocimetry (DPIV), and dynamic light scattering (DLS) [13]. Using optical imaging to monitor factors involved in ciliary dysfunction facilitates studies of disease mechanism, progression, and response to treatment. This review will present an overview of ciliary function, optical imaging modalities, and clinical applications of OCT in the study of ciliopathies.

## 2. Structure and Function of Cilia

### 2.1. Anatomy of Cilia: Structure and Classification

Cilia are organelles that are present on the surface of most cells in eukaryotes. The core of a cilium consists of an axoneme, which is a microtubule-based cytoskeletal structure covered by a specialized plasma membrane. The axoneme extends from the basal body, a microtubule-organizing center derived from centrioles, into the extracellular space [14,15]. Cilia are found on the surface of a variety of cell types and are classified into three basic categories: motile, primary, or nodal. Motile cilia have a highly ordered structure—the 9+2 axoneme, where nine interconnected microtubule doublets surround a central pair of microtubule singlets [16]. Each doublet has two rows of “dynein arms”, which contain ATP-powered motor proteins known as dyneins. Ciliary motility is generated by dynein-driven sliding of outer microtubule doublets relative to one another [4,17,18]. The central pair of microtubules is connected to the outer doublets by radial spokes and acts as a guiding structure to coordinate movement [19]. In contrast, primary cilia are non-motile with a 9+2 structure. They lack the central pair of microtubules needed to generate motile force and the dynein arms that make movement possible [14,20]. The third class, nodal cilia, have dynein arms and a 9+2 axoneme. As they lack radial spokes and a central pair of microtubules, they have a rotary movement rather than a coordinated whip-like motion seen with motile cilia [20].

### 2.2. Physiological Roles of Cilia

Cilia are essential structures that contribute to various physiological processes across multiple systems. During embryonic development, cilia-driven fluid flow plays a role in establishing the left–right axis of the body. The coordinated movement of fluid within the embryonic node, facilitated by cilia, helps establish asymmetric signaling patterns that direct the proper positioning of organs [21,22]. Additionally, cilia are crucial for the development of the inner ear and central nervous system. In the developing inner ear, cilia are involved in the formation and maintenance of the structures responsible for hearing and balance [21,23]. In the central nervous system, ependymal cilia line the ventricles of the brain and help regulate the flow of cerebrospinal fluid (CSF), which is critical for neural development and the transport of new neurons [21,24].

Cilia facilitate the transport and movement of substances within the body by beating in an orchestrated wavelike fashion [14]. In the respiratory system, ciliated epithelial cells beat in metachronal waves, generating directional fluid flow to clear mucus and debris from the airways [25,26]. This coordinated action, known as mucociliary clearance, is the primary innate defense mechanism of the lungs [27]. Similarly, in the fallopian tubes, the synchronized beating of cilia lining the luminal epithelium generates the movement of fluid and ovum through the oviduct [28]. Motile cilia also line cerebral ventricles, generating localized CSF flow. The maintenance of normal flow is essential for brain homeostasis, toxin clearance, and the transport of signaling molecules [24].

Non-motile cilia are involved in signal transduction, acting as sensors that detect and transmit mechanical and chemical stimuli. The ciliary membrane is rich in receptors and ion channel proteins that initiate signaling pathways [29]. Notably, cilia play a critical role in the activation of the Sonic Hedgehog (Shh) signaling pathway, which is involved in cell differentiation, migration, and growth during development and adulthood. Shh signaling, mediated by cilia, regulates the patterning of tissues, organ development, and stem cell function [30]. Growth factors and other signaling molecules that are transported through the ciliary membrane activate intracellular cascades that control various aspects of cellular behavior, from motility to differentiation [29].

### 2.3. Imaging of Cilia

Although advances have been made in establishing the essential role of cilia, further studies of ciliary activity are needed to understand how ciliary dysfunction can lead to developmental pathologies and severe disease. Studying the anatomy of cilia is performed at a nanoscopic scale. The microtubules that make up cilia are about 25 nm wide [31]. Transmission electron microscopy (TEM) has the requisite resolution to examine features including the microtubular arrangement and the dynein arms that drive ciliary motion. However, sample fixation is needed for TEM preparation, which may mechanically or chemically disturb the function of ciliated cells. The fixation process precludes the observation of ciliary movement in real time, making it an unsuitable method for studying ciliary dynamics [3,32].

#### 2.3.1. Ciliary Beat Frequency

In contrast to the static analysis offered by TEM, functional imaging techniques enable the study of ciliary motion and behavior. An important metric in ciliary function is the CBF, the tempo at which each individual cilium beats. Each cilium typically extends approximately 10 µm in length and beats at a rhythmic pace [3]. Measuring the CBF involves monitoring the movement of the ciliary stalk and does not require the high resolution needed for studying the ultrastructure [25,33]. Commonly used modalities are the photomultiplier and the photodiode, which indirectly measure the CBF by detecting fluctuations in the intensity of light passing through and scattered by beating cilia [25,33]. Another method, using white light or differential interference contrast (DIC) microscopy combined with high-speed video imaging, resolves the actual cilia themselves. Samples can be viewed immediately under a microscope while a camera records at high speeds. This imaging allows the precise beat pattern to be viewed at reduced frame rates or a frame-by-frame rate [3,25,26]. Other forms of microscopy such as confocal fluorescence microscopy have also been used to directly capture cilia [34]. Confocal fluorescent images are based on fluorescent emissions from a specific area defined by the pinhole of the microscope. Cilia can be fluorescently labeled and directly visualized to quantify the CBF. In most confocal setups, one focal point is recorded at a time, and it is scanned in the xy-plane (en face) and then along the *z*-axis (depth) to capture the full volume. While xy-plane scanning can be conducted without moving the sample or objective, *z*-axis scanning typically requires mechanical movement of the objective or the sample, often using a piezoelectric element, which can potentially disturb the sample [3].

#### 2.3.2. Airway Surface Liquid and Periciliary Layer

Another important aspect of ciliary function to study is the flow generated by cilia. At length scales of approximately 10–100 µm, adjacent cilia beat in the same direction and in coordinated, metachronal waves to propel fluid [3]. In the respiratory tract, cilia are located in the ASL, the fluid covering the epithelial lining. The ASL is comprised of the periciliary layer PCL, a watery layer that surrounds the cilia, and the mucus layer (ML), a viscoelastic layer rich in mucins directly above the PCL. The PCL lubricates the mucus and keeps it at an optimal distance from the cilia to allow for mucus clearance by ciliary beating [35,36]. The flow generated by cilia is characterized by a low Reynolds number and is described as microfluidic flow [37]. Imaging microfluidic flow requires a spatial resolution of 1–10 µm and a relatively large field of view of at least 1 mm. Imaging modalities, such as light, epifluorescence, and confocal microscopy satisfy these criteria. These techniques can be combined with methods for tracking velocimetry, such as dye tracing, particle tracking, digital particle imaging, speckle tracking, and light scattering [3].

#### 2.3.3. Mucociliary Transport and Bulk Flow

The coordinated interaction of the airway surface layer components generates microfluidic flows that coalesce to create the bulk transport of mucus, known as mucociliary clearance (MCC). This process occurs over larger length scales of sub-mm to cm diameters [3,27]. Understanding these complex flows requires integrating knowledge across multiple scales—from the microscopic behavior of individual cilia to the macroscopic transport of mucus across large sections of the airway. The most common method to measure MCC on a macroscopic scale is two-dimensional scintigraphy, by which the movement of inhaled radioisotopes is tracked with a gamma camera. Other methods, such as PET imaging, CT tracking of tantalum discs [38], fluorescent microscopy [39], and timing transit of saccharin in the nasal sinuses [2] have also been used.

The ability to study these parameters of ciliary function can help elucidate both physiological and pathogenic processes. The currently used methods of investigation have proven useful for identifying individual cilia, assessing beating patterns, and studying one part of the whole mechanism of ciliary activity [40]. However, to better investigate interactions between the elements the simultaneous capture of global ciliary activity and flow dynamics is required.

## 3. Optical Imaging

### 3.1. General Principles, Advantages, and Limitations of Optical Imaging

The development of imaging technologies has provided ways to understand pathogenesis and improve diagnosis and clinical management [41]. Optical imaging, which refers to a set of techniques that use light to capture high-resolution images of biological tissues and processes [42], has become pivotal in understanding cilia structure, function, and disease. Optical imaging methods have high spatial resolutions of micrometers or more, allowing for the detailed viewing of cellular structures and micro-anatomical features [41]. They also have high frame rates, acquiring serial images at more than ten frames per second. This real-time imaging makes it possible to study dynamic processes in living tissues [7]. Quantitative functional imaging, in the context of optical imaging, uses light to measure and visualize physiological changes, providing numerical data on the function of specific tissues or cells. More precise analysis and comparison across different conditions or time points can be achieved, often through the use of fluorescent probes that selectively target specific biological processes [43]. Unlike other modalities, such as CT or PET that use ionizing radiation, optical imaging uses visible or near-infrared light, which is considered non-invasive and safe [44].

While currently used methods can capture specific features of ciliary function, there are some limitations. Optical microscopy can resolve individual cilia movements, and high-speed digital camera microscopy can record the entire beating cycle. However, a conventional digital camera system cannot be used in vivo, thus limiting measurements in physiological settings [45]. Studying metachronal waves in vivo requires both cellular resolution and a high imaging speed. Thus, most studies use nasal brush sampling, cell culturing, or movement tracking of radiolabeled markers, which are invasive, slow to process, or require bulky equipment [46]. Additionally, no single technique can perform all the measurements of ciliary activity [47].

### 3.2. Epifluorescence, Confocal, and Phase-Contrast Microscopy

Epifluorescence microscopy involves the excitation of a sample and the imaging of the emitted fluorescence with a camera. A typical epifluorescence illumination compound microscope has a resolution of 200 nm. As the whole sample is illuminated, both in-focus and out-of-focus light are detected. Thus, it may be difficult to resolve the spatial relationship between two probes without further analysis. Additionally, as depth information cannot be obtained, conclusions about volumes cannot be made [3].

Confocal microscopy uses a laser to illuminate a single spot on a slide, with fluorophores emitted through a pinhole and detected by a photomultiplier tube. The tube then relays the information as an electrical signal. The use of lasers for illumination increases the resolution to ~2–3 nm. Additionally, the use of a pinhole blocks scattered and out-of-focus light before it arrives at the detector and restricts imaging to one focal plane. This optical sectioning increases resolution and provides information about depth. This method reduces background noise and image blurring compared to epifluorescence microscopy, which uses uniform and indiscriminate illumination. However, confocal imaging is slower and less accessible for many researchers [48,49].

Although these methods can provide clear images and are straightforward to segment and evaluate, labeling using a fluorescent dye or protein can affect cellular morphology. Phase-contrast microscopy is an alternative that does not damage cells and can be performed quickly. This technique involves converting phase shifts in light passing through a sample into observable changes in light amplitude. However, imperfections in the conversion process result in frequent imaging artifacts, which hinder the automated processing of images due to the challenge of segmenting cells from the background [50,51].

### 3.3. Optical Coherence Tomography

Optical coherence tomography (OCT) offers a promising alternative. OCT is an interferometry-based, non-contact technique that uses broadband laser light, which is reflected by the components of a structure of interest. Analogous to ultrasound, OCT measures echo delay in backscattered signals but uses light rather than sound. Since the natural reflectance of light back-scattered from a sample determines the contrast, exogenous dyes or contrast agents are not needed [6,26,52]. This method uses non-ionizing infrared light that is safe for use in biological tissue and does not damage the epithelial cell layer. Thus, prolonged investigations of tissues with minimal changes to the natural environment are possible [44,47,52]. Imaging is conducted with a micrometer scale resolution over a depth of several millimeters, or centimeters in low-scattering media, at relatively fast acquisition speeds (within seconds) [53,54]. OCT naturally performs optical sectioning, which allows for the isolation of the cilia layer. Simultaneous cross-sectional structural imaging and functional live capture of ciliary movement can be obtained at a high depth resolution. Several key parameters, including the CBF, the ASL depth, the PCL depth, the ciliary stroke pattern, and the MCT rate, can be directly studied from the same series of images [47,55].

#### 3.3.1. Principle of Interferometry

Many advanced optical imaging methods, including OCT, employ the principle of interferometry. Light waves from two sources can interfere with each other, either amplifying or canceling each other out depending on their phase relationship [56]. A single, low-coherence light source is split into two beams: one is directed at the sample and another serves as a reference. The sample beam reflects off different layers within the specimen, while the reference beam is reflected off a mirror at a known distance and returns to the detector. The light reflected from the sample is then recombined with the reference beam, and the resulting interference pattern is recorded [41,57]. Even small changes in the sample’s optical properties, such as variations in the refractive index or surface structure, can alter the interference pattern, enabling high-precision measurements [56].

The basic setup for optical imaging systems using interferometry typically includes a light source, a beam splitter, the sample to be imaged, and a detector. For interferometric techniques like OCT, a broadband light source is used, emitting a range of wavelengths. The light is split into two parts by a beam splitter. The interference pattern created by the combined reflected light from the sample and the reference beam is measured by a photodetector. Reflected light from subsurface structures is collected, while photons that are diffusely scattered are disregarded, reducing the background signal [26]. The resulting data are processed to reconstruct an image of the sample with depth information [41,56]. This approach allows for non-invasive, in vivo imaging, making it especially valuable for visualizing subsurface structures in tissues.

#### 3.3.2. Types of Imaging Systems

There are two primary variants of OCT systems that differ in how they acquire and process depth information: time-domain OCT (TD-OCT) and frequency-domain OCT (FD-OCT).

Time-domain OCT operates by measuring the time delay between the reference beam and the sample’s reflected light. In TD-OCT, a single photodetector is used to measure the interference signal over time, and a moving reference mirror is typically used to scan through different depths of the sample. As the mirror moves, the time it takes for light to return from different depths is recorded, allowing for the generation of depth-resolved images. TD-OCT offers high axial resolution due to the use of broadband light, but it has limitations in terms of speed. The mechanical movement of the reference mirror can be slow, which makes the system less suited for applications requiring high-speed imaging, such as live tissue monitoring or intraoperative imaging [58,59].

Frequency-domain, or Fourier domain, OCT has improved sensitivity and imaging speeds compared with TD-OCT [60]. In FD-OCT, light from a tunable laser or broadband light source is directed at the sample, and the reflected light is collected by a spectrometer or a photodetector. There are two main configurations of frequency-domain OCT: spectral-domain OCT (SD-OCT), which captures the entire spectrum of light reflected from the sample in a single shot, and swept-source OCT (SS-OCT), which uses a tunable laser to rapidly scan through different wavelengths [61,62]. Faster data acquisition is achieved compared to TD-OCT, as the mechanical movement of a reference mirror is not required [54].

#### 3.3.3. Advanced Imaging Techniques

In the context of functional imaging to study fluid flow, various velocimetry techniques can be used with OCT, including Doppler, particle tracking velocimetry (PTV), digital particle image velocimetry (DPIV), and dynamic light scattering (DLS).

Doppler velocimetry is the most common type of flow measurement in OCT. [13] Doppler OCT (D-OCT) can produce high-resolution images while also detecting micromotion. D-OCT works by measuring changes in back-scattered light to determine velocity. D-OCT has already been used in clinical settings to detect blood flow and tissue vibrations. This modality can be used to detect the microscopic motion of cilia, even at moderate resolutions [44].

Doppler-OCT can only measure velocity along the axis by analyzing phase changes over time. PTV-OCT tracks the movement of tracer particles in 2D between frames, allowing it to measure both velocity components (*x*, *y*) in the plane. DPIV-OCT also measures these in-plane velocity components by looking at how objects shift between frames, but it does this by analyzing spatial correlations at displaced positions in an intermediate scattering state [63]. Images are divided into smaller regions of interest (ROIs), and the ROIs of adjacent frames are cross-correlated. By calculating the correlation function between regions and finding the location that maximizes the correlation, the displacement and velocity can be estimated. DPIV can also be applied to serial volumetric data to estimate 3D3C (three dimensional, three component—*x*, *y*, *z* axes) velocity [13]. Speckle tracking is similar, but it works in a dense scattering environment where individual particles cannot be resolved. DLS-OCT looks at how the signal changes over time due to the movement of densely packed, sub-resolution particles. Faster-moving particles cause the signal to change more quickly. If diffusion effects are small or accounted for, DLS-OCT can estimate the total speed of movement [63].

#### 3.3.4. Technical Considerations in Imaging of Cilia

Cilia are typically 0.2–10 μm in diameter, which requires high axial (depth) and lateral (cross-sectional) resolution to study their fine structure [3]. Axial resolution, which refers to the ability to resolve structures along the optical axis, is needed to distinguish cilia from adjacent tissue layers. Lateral resolution is crucial for defining the cross-sectional profile of cilia and their orientation relative to the surrounding cells or tissue [26]. Achieving the required level of resolution necessitates advanced systems capable of achieving sub-micron precision. However, this level of resolution is not always compatible with the tissue penetration required to image cilia located deep within biological samples.

A challenge during the imaging of cilia is optimizing the signal-to-noise ratio (SNR), which can be low when imaging cilia. The SNR refers to the ratio of the desired signal, which is the light scattered from ciliary structures, to the background noise, such as optical scattering from surrounding tissues or random variations in light intensity. Cilia have relatively low reflectivity compared to surrounding tissues, leading to weak backscattered signals. This low reflectivity reduces the SNR, making it difficult to distinguish cilia from the surrounding tissue. This limitation is particularly problematic in tissues with complex scattering properties [58,64].

Motion artifacts also pose a challenge in the imaging of cilia. Cilia are dynamic structures that move in a coordinated manner, performing oscillatory motions. Movement can also come from patient motion, respiratory cycles, or small vibrations from imaging equipment. This motion can cause blurring or distortion of the ciliary structure, making it difficult to resolve the intricate morphology and dynamics of cilia. Since OCT relies on precise measurements of light scattering to generate high-resolution cross-sectional images, even minor motion can introduce artifacts, which affects accurate analysis [58,65].

#### 3.3.5. Strategies to Improve Imaging of Cilia

Addressing these challenges through the development of advanced imaging techniques, system optimization, and post-processing methods has been crucial for enhancing imaging of cilia in both clinical and research applications.

To increase the resolution, different spectral ranges or the range of the wavelengths of light can be explored [66]. One laboratory developed a technique called micro-optical coherence tomography (μOCT). As a spectral-domain implementation of OCT, it uses ultra-broadband light sources with an engineered beam shape to produce 2 μm lateral resolution, 1 μm axial resolution, and 300 μm depth of focus imaging [67]. Another strategy to enhance resolution is the use of adaptive optics (AO). AO compensates for distortions introduced by optical aberrations, such as those caused by the sample’s refractive elements or tissue scattering. By adjusting the optical path of light, AO can correct these imperfections in real time, increasing the resolution. This is beneficial for imaging small structures like cilia, where high spatial resolution is essential to discern individual ciliary components and their interactions with surrounding cellular structures [53].

To improve the imaging speed, spectral-encoded interferometric microscopy (SEIM) was introduced. This system uses a high-speed swept-source laser as the light source rather than a continuous wave laser. Transmission diffraction grating scans the sample and detects light reflected from multiple positions simultaneously. As the light source sweeps through a range of wavelengths in milliseconds, higher frame rates, and thus faster data acquisition, can be achieved. This capability makes these systems ideal for real-time, high-speed imaging, such as in dynamic biological processes or fast-moving targets [28,46].

Image processing algorithms can improve the quality and utility of optical images by enhancing image contrast, reducing noise, and providing more accurate segmentation of ciliary structures from background tissue. Motion-correction algorithms can be used to track and compensate for ciliary movement during imaging sessions [65]. Post-processing algorithms can provide substantial noise reduction and outlier rejection [46,68]. With the large amount of image data that can be collected in real time, processing algorithms can also be used to handle large data sets [66].

Finally, the development of customized OCT probes enables more precise and targeted imaging of cilia. These probes can be tailored to optimize resolution, sensitivity, and depth of penetration for the imaging of cilia in different environments, such as within the airway. Probes can also be miniaturized for use in clinical settings, allowing for in vivo imaging of cilia in patients [69]. A relatively recent advance is the inclusion of a micro-motor at the distal tip of an OCT endoscope catheter. This setup prevents rotational scans from being affected by kinks in the probe, thus reducing image artifacts [66]. Another variation improves lateral resolution. As tightly focused light diverges, cross-sectional imaging at high resolution and over large depths becomes more difficult. By introducing a cylindrical waveguide for light to transmit through before propagation, multiple light propagation modes are produced, broadening the focus and improving the depth of focus [70].

## 4. Applications of Optical Imaging of Cilia

### 4.1. Cilia Dynamics

Using OCT to accurately and quantitatively study the airway microanatomy has been validated in comparison to gold standard techniques. As OCT provides high-resolution, non-invasive, real-time imaging without the need for dyes or contrast agents, this modality is well suited for ex vivo and in vivo applications. This technique has thus been applied to investigate the cilia structure and function in primary cell cultures, ex vivo animal and human respiratory tissues, and in vivo animal and human respiratory tracts [12,26,44]. The understanding of the role of cilia in human disease has benefited substantially from the availability and use of cell cultures and animal models [14].

#### 4.1.1. Ex Vivo Animal Models

An early study in 1989 demonstrated that optical imaging could provide information on ciliary movement. Laser light scattering spectroscopy was used to assess CBF in bovine trachea. The fluctuations in the intensity of light scattered from moving cilia were directly correlated with the activity of tracheobronchial cilia. The results indicate that light spectroscopy can be used to monitor the rate and efficiency of mucociliary clearance [71]. The 1 μm-resolution OCT (μOCT) techniqye has also been used to study CBF and MCT rates through rapid scanning to generate cross-sectional, functional imaging over a two-dimensional plane [12]. Another method, Doppler OCT, was used for the quantitative measurement of CBF without the need to resolve individual cilia. After capturing the movement of cilia, fast Fourier transform was performed to convert the time-based data into frequency data to determine how fast the cilia beat [44]. How CBF at different regions of the respiratory tract is affected by factors such as different temperatures or therapeutic drugs has been quantified using swept-source Doppler OCT [47].

Functional dynamics of cilia can be captured with spectral domain optical coherence microscopy (SD-OCM). Phase images of cilia measure changes in the optical path length, which determines the frequency and direction of ciliary movement [1]. Temporal and spatial ciliary motion patterns and the resulting propagation of metachronal waves can be studied with this high-speed, high-resolution system [45]. Another study using a spectrally encoded interferometric microscope was used to image ciliary metachronal wave propagations in excised rabbit trachea. Phase-resolved enhanced dynamic (PHRED) analysis conducted on these images showed the propagation direction of metachronal waves and the amplitude and frequency of cilia beating at each spatial location. By using PHRED analysis on raw images, the ciliary movement could be seen more distinctively [46].

Complex three-dimensional microfluidic flow can be quantified using OCT-based velocimetry methods. Digital particle image velocimetry (DPIV) OCT has been used to quantify the vectorial flow field of cilia in mouse trachea [13]. Flow is measured by using cross-correlation to compare how much mucus has moved between two frames or images [72]. Particle streak velocimetry–OCT can also be used to study cilia-driven fluid flow. PSV-OCT captures the streaks in space of tracer particles in sparsely seeded fluid flow. With maps of microscale flow velocity fields, the speed, direction, and patterns of flow can be determined [63].

#### 4.1.2. In Vivo Animal Models

Capturing anatomy and functional dynamics in vivo has also been performed in some studies. An early study demonstrated the use of laser light scattering spectroscopy in measuring ciliary movement. Laser light was delivered by flexible optical fibers during a bronchoscopy in swine. Based on the intensity of light scattered from beating cilia, the CBF could be obtained [71]. Quantifying CBF and cilia-driven flow can also be performed using full-field reflection interferometric confocal microscopy (FFICM). Flow in the immediate vicinity of a single ciliated cell was captured and used to study how patterning of ciliated cells influences bulk flow patterns [73]. A portable, endoscopic, swept-source anatomical optical coherence tomography (aOCT) system was used to capture local airway dynamics during respiration in a pig under paralysis. The luminal cross-sectional area measurements that were obtained with aOCT were confirmed to be accurate relative to CT scans [74].

#### 4.1.3. Ex Vivo Cell Cultures and Human Models

Advancements in optical imaging have provided insight into the functional microanatomy of human respiratory epithelia with improved resolution. μOCT, with its 1-micron resolution, has been used to examine airway surface dynamics, such as ASL, PCL, MCT, ciliary stroke patterns, and CBF in primary human bronchial epithelial cells (hBECs) [5]. In another study, OCT was used to observe the circular, hurricane-like mucus movement in hBECs, providing a dynamic view of mucus transport [72].

As OCT can be used to obtain cross-sectional anatomic imaging and live motion capture of the entire MCC apparatus, the effects of pathophysiological variations in mucus on ciliary beating and mucus transport have been studied [55]. One study used μOCT to assess acquired MCC defects by evaluating ASL, PCL, and CBF in cells exposed to hypoxic conditions [75]. In another study, polarization-sensitive OCT was used to monitor mucociliary clearance and quantify mucus concentration in hBEC cultures treated with hypertonic saline [76]. These applications enhance the understanding of respiratory disease mechanisms and potential therapeutic interventions.

Cells within tissue can be difficult to image with microscopic OCT due to speckle noise and a lack of contrast, so one study used dynamic microscopic OCT (dmOCT). Dynamic contrast was used for the OCT imaging of nasal concha samples to capture the highly dynamic signal generated by the continuous beating of cilia. The CBF was then calculated from the recorded data [77]. In another study, polyester microspheres were used as contrast agents and CBF was determined by temporal frequency analysis. The ciliated epithelium was identified within OCT images by examining the speckle variance, and the flow rate and direction were obtained through particle tracking [40]. Further advancing the understanding of ciliary motion, one study used imaging-based analysis to detect the coordination of ciliary motion, or metachrony, in human tracheal tissue. μOCT was used to visualize the presence and proportion of epithelium in metachrony. ASL, PCL, CBF, and MCT in areas with and without metachrony were then compared [78].

#### 4.1.4. In Vivo Human Models

In one study, endo-microscopic OCT (emOCT) with a custom build graded-index-lens (GRIN) rigid endoscope was used for imaging nasal tissue. Due to the high lateral resolution and increased depth of field, structures not visible with common OCT devices, such as vessels and single cells, were clearly seen. The high imaging frame rates also allowed mucus transport to be quantified by tracking single flakes inside the mucus. EmOCT offers new options for simple, minimally invasive diagnoses [79].

### 4.2. Ciliopathy

Due to advances in optical equipment, optical detectors, light sources, interference technologies, and data acquisition and processing, the use of OCT has progressed from an imaging tool mainly used in research laboratories to a clinical tool in various areas of medicine [41]. The study of ciliopathic diseases has been greatly aided by imaging tools such as OCT. Several different diseases can result from genetic or acquired ciliopathies. Advances in imaging capabilities to assess cilia structure, function, and motion have improved our understanding of the underlying pathophysiology and the potential to evaluate diagnostic and therapeutic options [26].

#### 4.2.1. Primary Ciliary Dyskinesia

Primary ciliary dyskinesia (PCD) is a genetically heterogeneous disease characterized by cilia that are nonmotile or beat in a dyskinetic fashion [25]. As multiple genes are affected and the functional consequences vary, studies have tested the utility of optical imaging in understanding and categorizing disease processes.

The diagnosis and risk stratification of PCD remain challenging due to the emergence of new genes and mutations. One study demonstrated the effectiveness of μOCT imaging for quantitatively assessing ciliary motion and mucus transport (MCT) in PCD mutant mice. μOCT has a higher resolution and imaging speed compared to conventional OCT, allowing for the visualization of cilia on individual cells and ciliary stroke. Ciliary expression, beating, and pattern were assessed and abnormalities in ciliary motion were linked to defective MCT. The analysis measured parameters such as a motile cilia area, ESR, and ARC, and identified how specific mutations disrupt ciliary motility. The findings show that reduced MCT can result from various etiologies like the absence of motile cilia, reduced CBF, or abnormal stroke patterns, all of which can affect mucociliary clearance in the lungs [80]. Thus, μOCT could potentially help identify treatments most likely to be effective based on the ciliary motion abnormality, while also enabling the monitoring of how novel therapeutic treatments impact the mucociliary apparatus.

Another way to categorize disease phenotypes is based on various ultrastructural defects that cause different beat patterns. High-resolution digital high-speed video (DHSV) imaging captures the precise beat pattern of cilia in three different planes. Slow motion analysis of the imaging can show dyskinetic movement that would have been missed by conventional methods relying only on CBF measurement. The patterns observed in cilia with defects of inner dynein arms, outer dynein arms, or radial spokes can be compared. The association of certain beat patterns with each of the ultrastructural defects can then be used to improve diagnostic testing [81,82].

In addition to diagnostic improvement, optical imaging can also be used to understand disease processes. OCT-based particle tracking velocimetry (OCT-PTV) combines high-resolution cross-sectional imaging and velocity flow field measurements, and is well suited for quantifying changes in ciliary flow. Changes in flow near ciliated surfaces were investigated in Xenopus embryos in vivo to understand the effects of knockdown of dynein axonemal heavy chain 9 (dnah9), a protein responsible for the generation of movement and force in cilia [83]. Thus, OCT can be used to assess how genetic perturbations affect ciliary performance, elucidating disease mechanisms.

#### 4.2.2. Cystic Fibrosis

Cystic fibrosis (CF) is an autosomal-recessive genetic disorder caused by mutations in the CF transmembrane conductance regulator (CFTR) gene, which normally produces a secretory anion channel protein. Defective epithelial ion transport results in impaired mucociliary transport, infection, and a loss of pulmonary function [12,72,84]. The application of optical imaging techniques has provided a way to look at pathophysiological processes in a more specific way.

Visualizing the airway surface functional microanatomy to study components of the MCT process can be challenging due to the resolution needed (~1 μm). μOCT has the requisite resolution to see the ciliary stroke pattern, CBF, MCT, ASL, and PCL noninvasively. Studies conducted with excised tracheas of porcine and rat models showed the depletion of ASL and PCL, which resulted in the blunting of mucociliary transport [12]. μOCT with an intranasal probe in patients with CF showed the depletion of PCL, a loss of ciliation, elevated mucus reflectance intensity, and a delayed mucociliary transport rate [67,84]. The ability to look at morphological changes and functional impairment provides an improved understanding of pathophysiology. Additionally, acquiring quantitative metrics of airway function can enhance disease monitoring, which currently relies largely on spirometry and subjective reporting by patients due to the lack of direct monitoring tools [67].

Optical imaging can also play a role in the monitoring of therapeutic responses. A study using a cross-correlation method for speckle tracking quantified the flow of thick and optically turbid mucus in hBE cells [72]. As turbid fluids can be challenging to image, this method provided the ability to monitor improvements in MCC. Another study using mOCT in humans for the in vivo measurement of mucus transport velocity [79] offered a way to quantitatively evaluate a parameter before and after treatment. mOCT was used in mice to detect differences in response to different inhaled mucus-mobilizing interventions. The induction of bulk mucus transport and the increase in the mucus layer height were evaluated. This type of imaging can be used to study the effectiveness of therapies for patients with muco-obstructive lung diseases [85].

#### 4.2.3. Chronic Obstructive Pulmonary Disease

Impaired mucus clearance and the resulting airway mucus obstruction are critical abnormalities in chronic obstructive pulmonary disease (COPD) [85]. Possible reasons for MCC dysfunction include changes in mucus generation and composition, and cilia structure and function [86]. Optical imaging techniques have been used to investigate disease mechanisms and potential therapeutic strategies.

One study used μOCT on tracheal explants of ferrets chronically exposed to cigarette smoke to study functional microanatomy of the MCT apparatus, and particle tracking to determine mucus viscosity. The comparison of ciliary activity between controls and induced COPD helped elucidate mechanistic and pathophysiological changes [87].

Another study showed the use of high-speed μOCT in quantifying differences between therapeutic approaches to mobilize mucus in the trachea. The mean mucus layer height and mucus transport velocity were compared in vivo among mice subjected to isotonic saline, hypertonic saline, and bicarbonate [85]. Thus, optical imaging can detect changes to ciliary activity in response to different mucus-mobilizing interventions in vivo to facilitate the development of more effective therapies.

μOCT imaging on in vitro HBE cells and ex vivo human trachea was used to monitor changes in ASL depth and CBF with smoke exposure and after treatment with ivacaftor. Observing how smoke exposure affects ciliary function contributes to the understanding of COPD pathogenesis. The confirmation that ivacaftor treatment restores ciliary activity and ASL levels provides a possible treatment target to address mucus stasis in COPD. These studies demonstrate the utility of optical imaging in understanding disease mechanisms and evaluating potential therapeutic strategies [88].

#### 4.2.4. Chronic Sinusitis

The disruption of normal mucociliary clearance in the sinonasal epithelium is the underlying pathophysiologic process of chronic rhinosinusitis [75]. Studying the components of MCC can help establish a mechanistic basis for this disease. μOCT was used to image the medial and lateral walls of maxillary sinus tissue harvested from rabbits, in which chronic sinusitis was induced. While micro-computed tomography (CT) scanning can assess sinus opacification and endoscopy can be performed to examine the nasal cavity, μOCT has sufficient resolution to directly see and quantify microanatomic parameters, such as PCL, CBF, and MCT. μOCT imaging has an axial resolution of ~1 μm in tissue compared to traditional OCT imaging with an axial resolution of ~3 μm. This enables more precise PCL measurement, especially in disease conditions in which PCL is significantly reduced. Diminished mucociliary function with PCL depletion, decreased CBF, and severely delayed MCT was seen when compared to controls [89].

Another study also used μOCT imaging of human sinonasal epithelial (HSNE) cultures to evaluate these components. The mean ASL and PCL depth thickness were significantly decreased and the mean CBF was significantly slower. Acquiring these parameters required multiple devices in the past. However, OCT enables simultaneous capture at a resolution comparable with “gold standard” imaging techniques [75]. These studies support the use of optical imaging to investigate the disease process in chronic sinusitis.

#### 4.2.5. COVID-19

SARS-CoV-2 binds to its cellular receptor, angiotensin-converting enzyme 2 (ACE2), which has high expression in respiratory epithelial cells, and thereby disrupts mucociliary function. μOCT imaging on excised trachea from hamsters inoculated with SARS-CoV-2 showed a decrease in MCT, a loss of motile cilia, and abnormal ciliary beating in residual cilia [90]. Intranasal μOCT imaging performed on individuals with symptomatic COVID-19 showed mucus accumulation, denuded epithelium, and increased immune cell infiltration [91]. Mucociliary activity is a good predictor of mucosal function and can be used to assess respiratory function [2]. The ability to assess abnormal ciliated cell function can help in monitoring disease progression and determining disease severity. The previously used measure of viral loads has not consistently indicated prognostic potential for illness severity. OCT could be used to investigate pathogenic mechanisms and define functional parameters for disease stratification.

#### 4.2.6. Hyperoxia

Oxygen supplementation is essential in the treatment of respiratory failure; however, there is the potential for damage to the ciliated epithelium. Previous studies have demonstrated changes in respiratory epithelium after exposure to high levels of oxygen; however, the consequences of those changes on directional flow rates are not well understood. Optical imaging-based flow diagnostics can help determine the effects of hyperoxia on cilia-driven fluid flow. Particle tracking velocimetry optical coherence tomography (PTV-OCT) can track particles in both the transverse and axial directions in a ~100 to 1000 μm/s flow velocity range. In one study, PTV-OCT was used on excised mouse trachea to image cilia-driven fluid flow. There was a statistically significant decrease in average flow speed after exposure to hyperoxic conditions [92]. Thus, particle tracking using OCT imaging can detect flow perturbations.

#### 4.2.7. Congenital Hydrocephalus

Ependymal cells that line the brain ventricles have motile cilia, which are important for CSF production and flow through the cerebral aqueduct [93]. Altered CSF flow dynamics have been implicated in congenital hydrocephalus, but past methods have been insufficient for investigating the role of ciliary dysfunction [94]. OCT imaging in vivo of Xenopus tropicalis has been used to study CSF flow dynamics and ependymal cilia function. A series of 2D images were captured using OCT, and the movement of native free-floating particles was tracked across these images by comparing spatial positions over time. The CSF flow map generated using this particle tracking method with intraventricular particles showed multiple polarized flow fields throughout the ventricular system. Having a way to visualize CSF flow and analyze the subcellular architecture involved in cilia polarization can provide new insights into how flow fields are generated and how they change in disease states [94,95].

### 4.3. Future Directions

Despite its potential, there are obstacles that limit the widespread use of OCT in clinical practice. One barrier is the high costs of this technology, particularly for smaller practices or those in resource-limited settings. Additionally, specialized training and expertise is needed for the operation of the equipment, the proper interpretation of imaging, and integration into clinical decision-making. The time required for the imaging and subsequent analysis of OCT scans can be an obstacle in busy clinical environments, where rapid, real-time interpretation is preferred, particularly in emergency or routine settings [96].

As the advantages of OCT outweigh its disadvantages, overcoming current limitations to expand clinical utility is an important next step. One area of development is the miniaturization of OCT devices to facilitate in vivo imaging [12]. Smaller, portable OCT systems could make it easier to use in a wider variety of clinical settings and reduce costs. Automated analysis of OCT images through machine learning algorithms and artificial intelligence could reduce the need for specialized expertise and improve the speed and accuracy of diagnostics. Advances in OCT technology to acquire faster scans with fewer artifacts and reach deeper tissues, through adaptive optics and longer-wavelength OCT, increase clinical utility [53,96].

## 5. Conclusions

Cilia-driven fluid flow is critical in many physiological processes. Ciliopathies thus have diverse and often debilitating effects on multiple organ systems. Non-invasive methods of investigation can aid in the medical management of diseases. Proper imaging reduces the need for invasive exploratory procedures and trial-and-error approaches before determining a treatment approach. Optical imaging, in particular OCT, is well suited for the study of cilia structure and function, offering non-invasive, high-resolution, real-time imaging. Combined with various velocimetry methods, OCT can be used for quantitative studies of cilia-generated microfluidic flow. Current research highlights its potential in diagnosing ciliopathies, monitoring disease progression, and evaluating the efficacy of therapeutic interventions.

The ongoing development of OCT technology, including advances in resolution, contrast, and speed, holds significant promise for expanding its application in ciliopathy research. Furthermore, combining OCT with complementary imaging modalities may offer a more comprehensive understanding of cilia’s role in health and disease. Continued innovation in imaging techniques, coupled with a deeper understanding of cilia biology, will aid in early detection, personalized treatment planning, and real-time monitoring of ciliopathies.

## Data Availability

No new data were created or analyzed in this study.

## References

[1] Ansari R., Buj C., Pieper M., König P., Schweikard A., Hüttmann G. (2015). Micro-anatomical and functional assessment of ciliated epithelium in mouse trachea using optical coherence phase microscopy. Opt. Express.

[2] Koparal M., Kurt E., Altuntas E.E., Dogan F. (2021). Assessment of mucociliary clearance as an indicator of nasal function in patients with COVID-19: A cross-sectional study. Eur. Arch. Otorhinolaryngol..

[3] Huang B.K., Choma M.A. (2014). Microscale imaging of cilia-driven fluid flow. Cell Mol. Life Sci..

[4] Afzelius B. (2004). Cilia-related diseases. J. Pathol..

[5] Liu L., Chu K.K., Houser G.H., Diephuis B.J., Li Y., Wilsterman E.J., Shastry S., Dierksen G., Birket S.E., Mazur M. (2013). Method for Quantitative Study of Airway Functional Microanatomy Using Micro-Optical Coherence Tomography. PLoS ONE.

[6] Pang C., An F., Yang S., Yu N., Chen D., Chen L. (2020). In vivo and in vitro observation of nasal ciliary motion in a guinea pig model. Exp. Biol. Med..

[7] Kosaka N., McCann T.E., Mitsunaga M., Choyke P.L., Kobayashi H. (2010). Real-time optical imaging using quantum dot and related nanocrystals. Nanomedicine.

[8] Jayaraman S., Song Y., Vetrivel L., Shankar L., Verkman A.S. (2001). Noninvasive in vivo fluorescence measurement of airway-surface liquid depth, salt concentration, and pH. J. Clin. Investig..

[9] Griesenbach U., Soussi S., Larsen M.B., Casamayor I., Dewar A., Regamey N., Bush A., Shah P.L., Davies J.C., Alton E.W.F.W. (2011). Quantification of Periciliary Fluid Height in Human Airway Biopsies Is Feasible, but Not Suitable as a Biomarker. Am. J. Respir. Cell Mol. Biol..

[10] Ballard S.T., Trout L., Mehta A., Inglis S.K. (2002). Liquid secretion inhibitors reduce mucociliary transport in glandular airways. Am. J. Physiol.-Lung Cell. Mol. Physiol..

[11] Di Benedetto G., Magnus C.J., Gray P.T., Mehta A. (1991). Calcium regulation of ciliary beat frequency in human respiratory epithelium in vitro. J. Physiol..

[12] Shei R.-J., Peabody J.E., Rowe S.M. (2018). Functional Anatomic Imaging of the Airway Surface. Ann. Am. Thorac. Soc..

[13] Huang B.K., Gamm U.A., Bhandari V., Khokha M.K., Choma M.A. (2015). Three-dimensional, three-vector-component velocimetry of cilia-driven fluid flow using correlation-based approaches in optical coherence tomography. Biomed. Opt. Express.

[14] Davenport J.R., Yoder B.K. (2005). An incredible decade for the primary cilium: A look at a once-forgotten organelle. Am. J. Physiol.-Ren. Physiol..

[15] Beisson J., Wright M. (2003). Basal body/centriole assembly and continuity. Curr. Opin. Cell Biol..

[16] Satir P., Christensen S.T. (2007). Overview of Structure and Function of Mammalian Cilia. Annu. Rev. Physiol..

[17] Porter M.E., Sale W.S. (2000). The 9 + 2 Axoneme Anchors Multiple Inner Arm Dyneins and a Network of Kinases and Phosphatases That Control Motility. J. Cell Biol..

[18] Cooper G.M. (2000). Microtubule Motors and Movements. The Cell: A Molecular Approach.

[19] Satir P., Heuser T., Sale W.S. (2014). A Structural Basis for How Motile Cilia Beat. Bioscience.

[20] Hua K., Ferland R.J. (2018). Primary cilia proteins: Ciliary and extraciliary sites and functions. Cell Mol. Life Sci..

[21] Jonas S., Bhattacharya D., Khokha M.K., Choma M.A. (2011). Microfluidic characterization of cilia-driven fluid flow using optical coherence tomography-based particle tracking velocimetry. Biomed. Opt. Express.

[22] Okada Y., Takeda S., Tanaka Y., Belmonte J.-C.I., Hirokawa N. (2005). Mechanism of nodal flow: A conserved symmetry breaking event in left-right axis determination. Cell.

[23] Colantonio J.R., Vermot J., Wu D., Langenbacher A.D., Fraser S., Chen J.-N., Hill K.L. (2009). The dynein regulatory complex is required for ciliary motility and otolith biogenesis in the inner ear. Nature.

[24] Deng S., Gan L., Liu C., Xu T., Zhou S., Guo Y., Zhang Z., Yang G.-Y., Tian H., Tang Y. (2023). Roles of Ependymal Cells in the Physiology and Pathology of the Central Nervous System. Aging Dis..

[25] Chilvers M., O’Callaghan C. (2000). Analysis of ciliary beat pattern and beat frequency using digital high speed imaging: Comparison with the photomultiplier and photodiode methods. Thorax.

[26] Peabody J.E., Shei R.-J., Bermingham B.M., Phillips S.E., Turner B., Rowe S.M., Solomon G.M. (2018). Seeing cilia: Imaging modalities for ciliary motion and clinical connections. Am. J. Physiol. Lung Cell Mol. Physiol..

[27] Bustamante-Marin X.M., Ostrowski L.E. (2017). Cilia and Mucociliary Clearance. Cold Spring Harb. Perspect. Biol..

[28] He Y., Qu Y., Jing J.C., Chen Z. (2019). Characterization of oviduct ciliary beat frequency using real time phase resolved Doppler spectrally encoded interferometric microscopy. Biomed. Opt. Express.

[29] Christensen S.T., Pedersen L.B., Schneider L., Satir P. (2007). Sensory Cilia and Integration of Signal Transduction in Human Health and Disease. Traffic.

[30] Anvarian Z., Mykytyn K., Mukhopadhyay S., Pedersen L.B., Christensen S.T. (2019). Cellular signalling by primary cilia in development, organ function and disease. Nat. Rev. Nephrol..

[31] Fawcett D.W., Porter K.R. (1954). A study of the fine structure of ciliated epithelia. J. Morphol..

[32] Rezaei M., Soheili A., Ziai S.A., Fakharian A., Toreyhi H., Pourabdollah M., Ghorbani J., Karimi-Galougahi M., Mahdaviani S.A., Hasanzad M. (2022). Transmission electron microscopy study of suspected primary ciliary dyskinesia patients. Sci. Rep..

[33] Dalhamn T., Rylander R. (1962). Frequency of Ciliary Beat measured with a Photo-sensitive Cell. Nature.

[34] Doyle R.T., Moninger T., Debavalya N., Hsu W.H. (2006). Use of confocal linescan to document ciliary beat frequency. J. Microsc..

[35] Roomans G.M., Kozlova I., Nilsson H., Vanthanouvong V., Button B., Tarran R. (2004). Measurements of airway surface liquid height and mucus transport by fluorescence microscopy, and of ion composition by X-ray microanalysis. J. Cyst. Fibros..

[36] Matsui H., Randell S.H., Peretti S.W., Davis C.W., Boucher R.C. (1998). Coordinated Clearance of Periciliary Liquid and Mucus from Airway Surfaces. J. Clin. Investig..

[37] Milana E., Zhang R., Vetrano M.R., Peerlinck S., De Volder M., Onck P.R., Reynaerts D., Gorissen B. (2020). Metachronal patterns in artificial cilia for low Reynolds number fluid propulsion. Sci. Adv..

[38] Sher A.C., Stacy M.R., Reynolds S.D., Chiang T. (2024). In vivo detection of pulmonary mucociliary clearance: Present challenges and future directions. Eur. Respir. Rev..

[39] Song E., Iwasaki A. (2020). Method for Measuring Mucociliary Clearance and Cilia-generated Flow in Mice by ex vivo Imaging. Bio-protocol.

[40] Ling Y., Yao X., Gamm U.A., Arteaga-Solis E., Emala C.W., Choma M.A., Hendon C.P. (2017). Ex vivo visualization of human ciliated epithelium and quantitative analysis of induced flow dynamics by using optical coherence tomography. Lasers Surg. Med..

[41] Popescu D.P., Choo-Smith L.-P., Flueraru C., Mao Y., Chang S., Disano J., Sherif S., Sowa M.G. (2011). Optical coherence tomography: Fundamental principles, instrumental designs and biomedical applications. Biophys. Rev..

[42] Dhawan A.P., D’Alessandro B., Fu X. (2010). Optical Imaging Modalities for Biomedical Applications. IEEE Rev. Biomed. Eng..

[43] Torigian D.A., Huang S.S., Houseni M., Alavi A. (2007). Functional Imaging of Cancer with Emphasis on Molecular Techniques. CA Cancer J. Clin..

[44] Lemieux B.T., Chen J.J., Jing J., Chen Z., Wong B.J.F. (2015). Measurement of ciliary beat frequency using Doppler optical coherence tomography. Int. Forum Allergy Rhinol..

[45] He Y., Jing J.C., Qu Y., Wong B.J., Chen Z. (2020). Spatial mapping of tracheal ciliary beat frequency using real time phase-resolved Doppler spectrally encoded interferometric microscopy. ACS Photonics.

[46] Miao Y., Jing J.C., Chou L., Zhu Z., Wong B.J.F., Chen Z. (2023). Phase-resolved dynamic wavefront imaging of cilia metachronal waves. Quant. Imaging Med. Surg..

[47] Jing J.C., Chen J.J., Chou L., Wong B.J.F., Chen Z. (2017). Visualization and Detection of Ciliary Beating Pattern and Frequency in the Upper Airway using Phase Resolved Doppler Optical Coherence Tomography. Sci. Rep..

[48] Elliott A.D. (2020). Confocal Microscopy: Principles and Modern Practices. Curr. Protoc. Cytom..

[49] Honzel E., Joshi A., Hernandez-Morato I., Pennington-FitzGerald W., Pitman M.J. (2024). A comparison of confocal and epifluorescence microscopy for quantification of RNAScope and immunohistochemistry fluorescent images. J. Neurosci. Methods.

[50] Yin Z., Kanade T., Chen M. (2012). Understanding the Phase Contrast Optics to Restore Artifact-free Microscopy Images for Segmentation. Med. Image Anal..

[51] Baručić D., Kaushik S., Kybic J., Stanková J., Džubák P., Hajdúch M. (2022). Characterization of drug effects on cell cultures from phase-contrast microscopy images. Comput. Biol. Med..

[52] Umezu K., Larina I.V. (2023). Optical coherence tomography for dynamic investigation of mammalian reproductive processes. Mol. Reprod. Dev..

[53] Pircher M., Zawadzki R.J. (2017). Review of adaptive optics OCT (AO-OCT): Principles and applications for retinal imaging. Biomed. Opt. Express.

[54] Volgger V., Sharma G.K., Jing J., Peaks Y.-S.A., Loy A.C., Lazarow F., Wang A., Qu Y., Su E., Chen Z. (2015). Long-range Fourier domain optical coherence tomography of the pediatric subglottis. Int. J. Pediatr. Otorhinolaryngol..

[55] Liu L., Shastry S., Byan-Parker S., Houser G., Chu K.K., Birket S.E., Fernandez C.M., Gardecki J.A., Grizzle W.E., Wilsterman E.J. (2014). An Autoregulatory Mechanism Governing Mucociliary Transport Is Sensitive to Mucus Load. Am. J. Respir. Cell Mol. Biol..

[56] Patel R., Achamfuo-Yeboah S., Light R., Clark M. (2014). Widefield two laser interferometry. Opt. Express.

[57] Pitris C., Brezinski M.E., Bouma B.E., Tearney G.J., Southern J.F., Fujimoto J.G. (1998). High Resolution Imaging of the Upper Respiratory Tract with Optical Coherence Tomography. Am. J. Respir. Crit. Care Med..

[58] Zhang J., Mazlin V., Fei K., Boccara A.C., Yuan J., Xiao P. (2023). Time-domain full-field optical coherence tomography (TD-FF-OCT) in ophthalmic imaging. Ther. Adv. Chronic Dis..

[59] Aumann S., Donner S., Fischer J., Müller F., Bille J.F. (2019). Optical Coherence Tomography (OCT): Principle and Technical Realization. High Resolution Imaging in Microscopy and Ophthalmology: New Frontiers in Biomedical Optics.

[60] Vakoc B.J., Yun S.H., de Boer J.F., Tearney G.J., Bouma B.E. (2005). Phase-resolved optical frequency domain imaging. Opt. Express.

[61] Boudoux C., Yun S.H., Oh W.Y., White W.M., Iftimia N.V., Shishkov M., Bouma B.E., Tearney G.J. (2005). Rapid wavelength-swept spectrally encoded confocal microscopy. Opt. Express.

[62] Yelin D., White W.M., Motz J.T., Yun S.H., Bouma B.E., Tearney G.J. (2007). Spectral-domain spectrally-encoded endoscopy. Opt. Express.

[63] Zhou K.C., Huang B.K., Gamm U.A., Bhandari V., Khokha M.K., Choma M.A. (2016). Particle streak velocimetry-optical coherence tomography: A novel method for multidimensional imaging of microscale fluid flows. Biomed. Opt. Express.

[64] Uribe-Patarroyo N., Post A.L., Ruiz-Lopera S., Faber D.J., Bouma B.E. (2020). Noise and bias in optical coherence tomography intensity signal decorrelation. OSA Contin..

[65] Scholler J. (2019). Motion artifact removal and signal enhancement to achieve in vivo dynamic full field OCT. Opt. Express.

[66] Gora M.J., Suter M.J., Tearney G.J., Li X. (2017). Endoscopic optical coherence tomography: Technologies and clinical applications. Biomed. Opt. Express.

[67] Chu K.K., Unglert C., Ford T.N., Cui D., Carruth R.W., Singh K., Liu L., Birket S.E., Solomon G.M., Rowe S.M. (2016). In vivo imaging of airway cilia and mucus clearance with micro-optical coherence tomography. Biomed. Opt. Express.

[68] Tang T., Deniz E., Khokha M.K., Tagare H.D. (2019). Gaussian process post-processing for particle tracking velocimetry. Biomed. Opt. Express.

[69] Cui D., Chu K.K., Yin B., Ford T.N., Hyun C., Leung H.M., Gardecki J.A., Solomon G.M., Birket S.E., Liu L. (2017). Flexible, high-resolution micro-optical coherence tomography endobronchial probe toward in vivo imaging of cilia. Opt. Lett..

[70] Yin B., Hyun C., Gardecki J.A., Tearney G.J. (2017). Extended depth of focus for coherence-based cellular imaging. Optica.

[71] Svartengren K., Wiman L.G., Thyberg P., Rigler R. (1989). Laser light scattering spectroscopy: A new method to measure tracheobronchial mucociliary activity. Thorax.

[72] Oldenburg A.L., Chhetri R.K., Hill D.B., Button B. (2012). Monitoring airway mucus flow and ciliary activity with optical coherence tomography. Biomed. Opt. Express.

[73] Sencan I., Huang B.K., Bian Y., Mis E., Khokha M.K., Cao H., Choma M. (2016). Ultrahigh-speed, phase-sensitive full-field interferometric confocal microscopy for quantitative microscale physiology. Biomed. Opt. Express.

[74] Balakrishnan S., Bu R., Iftimia N., Price H., Zdanski C., Oldenburg A.L. (2018). Combined anatomical optical coherence tomography and intraluminal pressure reveal viscoelasticity of the in vivo airway. J. Biomed. Opt..

[75] Tipirneni K.E., Grayson J.W., Zhang S., Cho D.-Y., Skinner D.F., Lim D.-J., Mackey C., Tearney G.J., Rowe S.M., Woodworth B.A. (2017). Assessment of acquired mucociliary clearance defects using micro-optical coherence tomography. Int. Forum Allergy Rhinol..

[76] Oeler K.J., Blackmon R.L., Kreda S.M., Robinson T., Ghelardini M., Chapman B.S., Tracy J., Hill D.B., Oldenburg A.L. (2024). In situ pulmonary mucus hydration assay using rotational and translational diffusion of gold nanorods with polarization-sensitive optical coherence tomography. J. Biomed. Opt..

[77] Kohlfaerber T., Pieper M., Münter M., Holzhausen C., Ahrens M., Idel C., Bruchhage K.-L., Leichtle A., König P., Hüttmann G. (2022). Dynamic microscopic optical coherence tomography to visualize the morphological and functional micro-anatomy of the airways. Biomed. Opt. Express.

[78] Lever J.E.P., Turner K.B., Fernandez C.M., Leung H.M., Hussain S.S., Shei R.-J., Lin V.Y., Birket S.E., Chu K.K., Tearney G.J. (2024). Metachrony drives effective mucociliary transport via a calcium-dependent mechanism. Am. J. Physiol.-Lung Cell. Mol. Physiol..

[79] Schulz-Hildebrandt H., Pieper M., Stehmar C., Ahrens M., Idel C., Wollenberg B., König P., Hüttmann G. (2018). Novel endoscope with increased depth of field for imaging human nasal tissue by microscopic optical coherence tomography. Biomed. Opt. Express.

[80] Solomon G.M., Francis R., Chu K.K., Birket S.E., Gabriel G., Trombley J.E., Lemke K.L., Klena N., Turner B., Tearney G.J. (2017). Assessment of ciliary phenotype in primary ciliary dyskinesia by micro-optical coherence tomography. JCI Insight.

[81] Chilvers M.A., Rutman A., O’Callaghan C. (2003). Ciliary beat pattern is associated with specific ultrastructural defects in primary ciliary dyskinesia. J. Allergy Clin. Immunol..

[82] Rossman C.M., Forrest J.B., Lee R.M.K.W., Newhouse M.T. (1980). The Dyskinetic Cilia Syndrome: Ciliary Motility in Immotile Cilia Syndrome. Chest.

[83] Huang B.K., Gamm U.A., Jonas S., Khokha M.K., Choma M.A. (2015). Quantitative optical coherence tomography imaging of intermediate flow defect phenotypes in ciliary physiology and pathophysiology. J. Biomed. Opt..

[84] Leung H.M., Birket S.E., Hyun C., Ford T.N., Cui D., Solomon G.M., Shei R.-J., Adewale A.T., Lenzie A.R., Fernandez-Petty C.M. (2019). Intranasal micro-optical coherence tomography imaging for cystic fibrosis studies. Sci. Transl. Med..

[85] Pieper M., Schulz-Hildebrandt H., Mall M.A., Hüttmann G., König P. (2020). Intravital microscopic optical coherence tomography imaging to assess mucus-mobilizing interventions for muco-obstructive lung disease in mice. Am. J. Physiol. Lung Cell Mol. Physiol..

[86] Hessel J., Heldrich J., Fuller J., Staudt M.R., Radisch S., Hollmann C., Harvey B.-G., Kaner R.J., Salit J., Yee-Levin J. (2014). Intraflagellar Transport Gene Expression Associated with Short Cilia in Smoking and COPD. PLoS ONE.

[87] Kaza N., Lin V.Y., Stanford D., Hussain S.S., Libby E.F., Kim H., Borgonovi M., Conrath K., Mutyam V., Byzek S.A. (2022). Evaluation of a novel CFTR potentiator in COPD ferrets with acquired CFTR dysfunction. Eur. Respir. J..

[88] Raju S.V., Lin V.Y., Liu L., McNicholas C.M., Karki S., Sloane P.A., Tang L., Jackson P.L., Wang W., Wilson L. (2017). The Cystic Fibrosis Transmembrane Conductance Regulator Potentiator Ivacaftor Augments Mucociliary Clearance Abrogating Cystic Fibrosis Transmembrane Conductance Regulator Inhibition by Cigarette Smoke. Am. J. Respir. Cell Mol. Biol..

[89] Cho D.-Y., Mackey C., Van Der Pol W.J., Skinner D., Morrow C.D., Schoeb T.R., Rowe S.M., Swords W.E., Tearney G.J., Woodworth B.A. (2018). Sinus Microanatomy and Microbiota in a Rabbit Model of Rhinosinusitis. Front. Cell Infect. Microbiol..

[90] Li Q., Vijaykumar K., Phillips S.E., Hussain S.S., Huynh N.V., Fernandez-Petty C.M., Lever J.E.P., Foote J.B., Ren J., Campos-Gómez J. (2023). Mucociliary transport deficiency and disease progression in Syrian hamsters with SARS-CoV-2 infection. JCI Insight.

[91] Vijaykumar K., Leung H.M., Barrios A., Fernandez-Petty C.M., Solomon G.M., Hathorne H.Y., Wade J.D., Monroe K., Slaten K.B., Li Q. (2022). COVID-19 Causes Ciliary Dysfunction as Demonstrated by Human Intranasal Micro-Optical Coherence Tomography Imaging. Am. J. Respir. Cell Mol. Biol..

[92] Gamm U.A., Huang B.K., Syed M., Zhang X., Bhandari V., Choma M.A. (2015). Quantifying hyperoxia-mediated damage to mammalian respiratory cilia-driven fluid flow using particle tracking velocimetry optical coherence tomography. J. Biomed. Opt..

[93] Ibañez-Tallon I., Pagenstecher A., Fliegauf M., Olbrich H., Kispert A., Ketelsen U.-P., North A., Heintz N., Omran H. (2004). Dysfunction of axonemal dynein heavy chain Mdnah5 inhibits ependymal flow and reveals a novel mechanism for hydrocephalus formation. Hum. Mol. Genet..

[94] Date P., Ackermann P., Furey C., Fink I.B., Jonas S., Khokha M.K., Kahle K.T., Deniz E. (2019). Visualizing flow in an intact CSF network using optical coherence tomography: Implications for human congenital hydrocephalus. Sci. Rep..

[95] Dur A.H., Tang T., Viviano S., Sekuri A., Willsey H.R., Tagare H.D., Kahle K.T., Deniz E. (2020). In Xenopus ependymal cilia drive embryonic CSF circulation and brain development independently of cardiac pulsatile forces. Fluids Barriers CNS.

[96] Zeppieri M., Marsili S., Enaholo E.S., Shuaibu A.O., Uwagboe N., Salati C., Spadea L., Musa M. (2023). Optical Coherence Tomography (OCT): A Brief Look at the Uses and Technological Evolution of Ophthalmology. Medicina.

